# A new perspective on hematological malignancies: m6A modification in immune microenvironment

**DOI:** 10.3389/fimmu.2024.1374390

**Published:** 2024-05-28

**Authors:** Shiyu Yang, Liping Xu, Haihui Zhuang, Fenglin Li, Ying Lu

**Affiliations:** ^1^ Department of Hematology, The Affiliated People’s Hospital of Ningbo University, Ningbo, China; ^2^ Institute of Hematology, Ningbo University, Ningbo, China

**Keywords:** m6A, hematological malignancies, immune microenvironment, immunotherapy, tumor treatment resistance

## Abstract

Immunotherapy for hematological malignancies is a rapidly advancing field that has gained momentum in recent years, primarily encompassing chimeric antigen receptor T-cell (CAR-T) therapies, immune checkpoint inhibitors, and other modalities. However, its clinical efficacy remains limited, and drug resistance poses a significant challenge. Therefore, novel immunotherapeutic targets and agents need to be identified. Recently, N6-methyladenosine (m6A), the most prevalent RNA epitope modification, has emerged as a pivotal factor in various malignancies. Reportedly, m6A mutations influence the immunological microenvironment of hematological malignancies, leading to immune evasion and compromising the anti-tumor immune response in hematological malignancies. In this review, we comprehensively summarize the roles of the currently identified m6A modifications in various hematological malignancies, with a particular focus on their impact on the immune microenvironment. Additionally, we provide an overview of the research progress made in developing m6A-targeted drugs for hematological tumor therapy, to offer novel clinical insights.

## 1 Introduction

Hematological malignancies, which arise from malignant clones of hematopoietic stem cells (HSCs), are a prevalent form of cancer with significant academic and clinical implications. The 7% global incidence each year and low probability of early detection and high recurrence rates make it a significant challenge in the field of medical research (1). Various therapeutic modalities have been employed in long-term hematology, including chemotherapy, radiation therapy, targeted therapy, hematopoietic stem cell transplantation, and cellular immunotherapy. Intricate immune evasion mechanisms play pivotal roles in the pathogenesis of hematological malignancies. Immunotherapies, particularly immune checkpoint inhibitors, T-cell immunotherapy, Chimeric Antigen Receptor T-Cell immunotherapy (CAR-T), and natural killer cell immunotherapy, have significantly prolonged the survival of patients with hematological malignancies (2). However, long-term survival statistics remain discouraging and immunotherapy has demonstrated limited efficacy in addressing hematological malignancies (3). One of the contributing factors to the limited efficacy of immunotherapy is immune evasion, and numerous studies have established a close correlation between the immune microenvironment, which serves as the ‘soil’ for tumor growth, and immune evasion (4). Therefore, to enhance the precision and efficacy of current immunotherapy approaches, it is crucial to investigate the regulatory mechanisms underlying immune evasion within the tumor microenvironment and identify biomarkers associated with successful immunotherapeutic outcomes. Epigenetic regulation, which encompasses DNA methylation changes, histone modifications, and RNA epigenetic regulation, has been shown to significantly influence numerous tumors (5, 6). The N6-Methyladenosine (m6A) modification, which is one of the most prevalent forms of RNA epigenetic modification, involves methylation at the nitrogen atom located at the sixth position of adenylate. Desrosiers discovered an m6A mutation in human mammalian Novikoff liver cancer cells in 1974 (7–10). Subsequent studies have established associations between m6A and various physiological processes, such as the circadian rhythm, heat shock response, neurodevelopment, and spermatogenesis (11–14). Additionally, m6A governs the activity of transcription factors, non-coding RNA molecules, and signaling pathways associated with carcinogenesis (15). Furthermore, it plays a pivotal role in regulating the tumor immune microenvironment. For instance, Du et al. meticulously examined alterations in m6A patterns across 255 myeloid leukemia specimens using 23 m6A regulators. Consensus clustering based on 23 m6A regulators revealed three distinct patterns of m6A changes that were largely consistent with the three immunophenotypes of malignancies (immune rejection, immunological activation, and immune inertia) (16). Furthermore, the m6A demethylase FTO can influence the efficacy of immunotherapy by regulating the expression of the immunological checkpoints PD-1/PD-L1 in melanoma (17, 18). Both these examples confirm the potential significance of m6A in shaping the immune microenvironment within tumors. This study provides an overview of the immunological milieu and biological role of m6A in hematological malignancies as well as possible treatment approaches.

## 2 Regulators of m6A methylation

**Figure 1:**
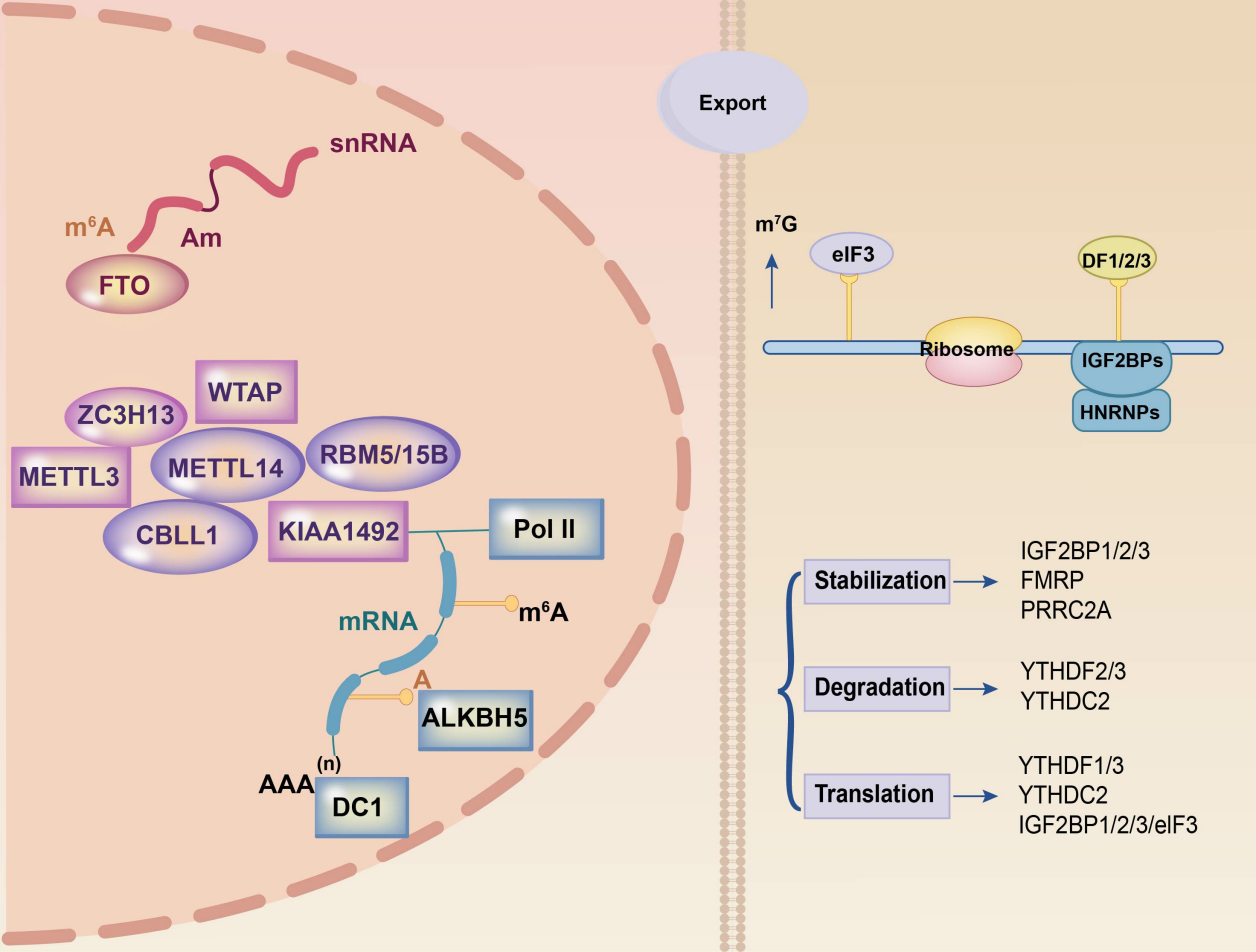
Summary of m6A modification machinery. The m6A methylation process on mRNA relies on three essential components: the writer, eraser, and reader. The writer complex, comprising METTL3 and METTL14 among others, is responsible for RNA methylation. FTO and ALKBH5 are part of the eraser complex that eliminates methylation modifications in the nucleus. The reader component, located in the cytoplasm and including YTHDF1, YTHDF2, and YTHDF3 among others, recognizes methylated mRNAs and facilitates post-transcriptional mRNA stabilization, translation, and degradation processes. These three components collaborate to orchestrate the entire m6A methylation process.

Three components control the reversible process of m6A methylation: the writer, eraser, and reader (19). The steady-state equilibrium of intracellular m6A levels was preserved by the interaction of these three components (**Figure 1**).

### 2.1 Writer

Methyltransferases, also known as writers, are a class of proteins that catalyze the synthesis of m6A. Bokar first discovered this class in 1994 (20). Methyltransferase 3 (METTL3), methyltransferase 14 (METTL14), methyltransferase 16 (METTL16), virlike m6A methyltransferase (Virma), and Wilms’ tumor 1-associating protein (WTAP), RNA binding motif protein 15 (RBM15), zinc finger CCCH domain-containing protein 13 (ZC3H13), and HAKAI are the primary components of the multi-component methyltransferase complex (MTC) (21–24). METTL3, a zinc-finger domain and methyl domain methyltransferase, is involved in catalysis. METTL14, which functions as a regulatory subunit, binds to METTL3 to create stable heterodimers and boosts catalysis. Reportedly, it exhibits no independent catalytic activity (25–27). WTAP catalyzes substrate RNA by generating a METTL3-METTL14-WTAP complex as a fundamental component of the MTC (28).

RBM15, ZC3H13, and HAKAI are key complex subunits. RBM15 binds to the METTL3/METTL14 dimer via WTAP, and knockdown results in decreased m6A expression (29). RBM15 has been identified as a possible prognostic biomarker for m6A in malignancies (30, 31). ZC3H13, however, binds WTAP, Virilizer, and HAKAI in the nucleus to assist the m6A process, and its knockout results in cytoplasmic translocation (32). Thus far, there is no evidence that HAKAI influences tumor growth via m6A.

### 2.2 Eraser

“Eraser” is the name given to m6A demethylase. As the name suggests, it is a key bridge in the dynamic and reversible process of m6A methylation. Obesity-associated protein (FTO) and AlkB homolog 5 (ALKBH5), which demethylate using the cofactors Fe2+ and α-ketoglutaric acid (33). FTO was the first m6A demethylase identified, and it regulates energy homeostasis and fat mass (34, 35). FTO is the predominant m6A eraser. Certain oncogenic proteins, including PML-RARA, FLT3-ITD, and NPM1, have been demonstrated to increase FTO expression. FTO expression increases the viability of human acute myeloid leukemia (AML) cells and blocks apoptosis (36). The ALKB family members ALKBH5 and FTO are iron- and 6-oxoglutarate-dependent nucleic acid oxygenases. m6A is a direct target of PD-L1 mRNA, and ALKBH5 regulates its expression. According to research, ALKBH5 deletion increases m6A in the 3’ UTR of PD-L1 mRNA and accelerates mRNA degradation in a YTHDF2-dependent way (37).

### 2.3 Reader

The m6A reading proteins, commonly referred to as “readers,” primarily encompass members of the YTH domain protein family (YTHDC1/2, YTHDF1/2/3), heterogeneous nuclear ribonucleoproteins (hnRNPs), insulin-like growth factor 2 mRNA-binding protein family (IGF2BP1/2/3), eukaryotic initiation factors, FMRP translation regulation protein 1, and leucine-rich pentapeptide repeat sequence proteins. Among these, YTHDC1 is the only intranuclear reader (38) that regulates nuclear RNA variable splicing, alternative polyadenylation, nuclear export, and degradation (39, 40). YTHDC1-mediated m6A alterations play critical regulatory roles in recognizing heterochromatin modification states and maintaining retrotransposon silencing (41). Furthermore, YTHDC2 functions as an RNA-deconjugating enzyme that enhances protein translation efficiency (14). The YTHDF protein family is associated with the stability of m6A-modified mRNAs. YTHDF2 is a reading protein that undergoes degradation. YTHDF2 is a complex protein with oncogenic and anti-tumor functions. YTHDF2 has an oncogenic role in most malignancies. By engaging eukaryotic translation initiation factor 3 (eIF3), which binds m6A-tagged mRNAs to ribosomes, YTHDF1 increases the translation of the cell cycle protein E2 (42). YTHDF3 increases mRNA translation and degradation (43). YTHDF3 works with YTHDF1 to increase mRNA translation and influences mRNA breakdown via YTHDF2-mediated methylation (44).

HNRNPA2/B1 recognizes the RGm6AC motif (the m6A core motif RGAC) and regulates selective shearing of RNA in a METTL3-dependent manner. Furthermore, they mediate and facilitate the processing of precursor miRNAs (45).

## 3 Relevance of m6A in the pathogenesis of hematological malignancies

### 3.1 The impact of m6A modifications on the pathogenesis of hematological malignancies

#### 3.1.1 AML and m6A

As the most frequent type of acute leukemia in adults, AML is characterized by the disruption of normal hematopoiesis and malignant clonal growth of a subset of myeloid cells (46). Several studies examined the association between m6A expression and acute myeloid leukemia (AML). In 2017, Vu et al. discovered that METTL3 was substantially expressed in AML cells, and that its expression level was adversely associated with the differentiation of normal myeloid cells. METTL3 knockdown in MOLM-13 cells causes the phosphorylation of serine/threonine protein kinase (PKB/AKT), which induces the differentiation and apoptosis of AML cells (47). Similarly, Barbieri et al. discovered that the CCAAT/enhancer-binding protein zeta (CZBPZ) attracted METTL3 to chromatin gene promoters, where it induced its deposition on related mRNA transcription factors (e.g., SP1 and SP2) and catalyzed the initiation of m6A modification (48). The pharmacological inhibition of METTL3 *in vivo* leads to prolonged survival in transplantation-impaired and various AML mouse models. This specifically targets key stem cell subpopulations in AML (49). Weng et al. further demonstrated that another m6A related methyltransferase, METTL14, was significantly expressed in AML cells, particularly in those with t (11q23), t (15;17), or t (8;21). This may be because METTL14 controls its mRNA targets (e.g., MYB and MYC) via m6A alterations to act an oncogenic roles (50). According to the findings of Sang et al. (51), METTL3 and METTL14 exert oncogenic functions in AML by increasing the amount of m6A in mdm2 mRNAs and targeting the mdm2/p53 signaling pathway. AML can be treated by increasing m6A levels in mdm2 mRNA and targeting the mdm2/p53 signaling pathway. Furthermore, Bansal et al. discovered that 32% of patients with AML had higher-than-normal m6A-related methyltransferase WTAP levels, which were substantially linked to specific molecular alterations, including NPM1 and FLT3-ITD (P < 0.05). WTAP knockdown reduced the phosphorylation of rapamycin-targeting protein (mTOR) and its downstream effector p70 ribosomal subunit 6 kinase, and mTOR dysregulation may be related to AML (52). In conclusion, the current study revealed that methyltransferases such as METTL3, METTL14, and WTAP are involved in AML cell proliferation and differentiation and are thus highly related to poor prognosis.

FTO, a key demethylase, is significantly expressed in AML cells, particularly in those with t (15;17)/PML-RARA, NPM1, and FLT3-ITD mutations and t(11q23)/MLL rearrangements. Both *in vivo* and *in vitro* experiments have shown that FTO blocks the normal functions of RARA and ASB2 in cellular differentiation and apoptosis by attenuating m6A levels within ASB2 and RARA mRNA transcripts, thereby promoting oncoprotein-driven cell transformation and AML progression (36). Furthermore, FTO enhanced CEBPA mRNA expression by modulating the m6A modification levels. Similarly, an increase in CEBPA acts on FTO to boost its transcription, resulting in a positive regulation. Besides, R-2-hydroxyglutarate (R-2HG) inhibits FTO, a competitive anabolic metabolite of hydroxyglutarate, which increases the abundance of m6A in AML cells and inhibits leukemia cell growth by downregulating CEBPA and FTO expression via the FTO/m6A/CEBPA signaling pathway (53).

In NPM1-mutant AML, the presence of NPM1-mA led to proteasomal degradation, thereby facilitating FTO upregulation. This subsequently activated the FTO/PDGFRB/ERK pathway and promoted AML cell division and proliferation. Therefore, FTO is envisaged as a promising new therapeutic target for NPM1-mutant AML (54). Furthermore, ALKBH5 has been demonstrated to be overexpressed in AML. Specifically, ALKBH5 promotes tumorigenesis in AML through post-transcriptional modulation of its main targets, such as TACC3, an oncogene linked to malignant prognosis. Through analysis of a database containing gene expression, prognosis, and genome-level alterations in AML cases, this study revealed that ALKBH5 was significantly overexpressed in various subtypes of AML patients and was strongly associated with poor prognosis in AML patients. Contrary to previous reports indicating a high frequency of deletions in the genomes of patients with AML, we found that the ALKBH5 gene has a low percentage of deletions in AML. It was postulated that ALKBH5 deletion not only did not reduce the ability of normal hematopoietic stem cells to replenish blood, but it also had a minor effect. However, a study conducted by Gao et al. revealed that deletion of ALKBH5 resulted in an abnormal accumulation of m6A modifications in RNA. This leads to the downregulation of RNA stability of metabolic enzyme-associated transcripts, such as Ogdh. Additionally, this accumulation causes an increase in the aberrant metabolite L-2-hydroxyglutarate (L-2-HG), which inhibits the tricarboxylic acid cycle energy generation process and hinders the adaptation of stem cells during stress hematopoiesis (55). Another study conducted using a mouse model showed that the deficiency of ALKBH5 leads to a moderate increase in the number of multiple progenitor cell populations and hindered the long-term self-renewal capacity of HSCs (56). Thus, the possibility of treating leukemia by targeting ALKBH5 remains unresolved and requires further research. Thus far, few studies have focused on m6A readers. Although YTHDF2 is not necessary for normal hematopoiesis, it contributes to leukemia development by shortening the half-life of the m6A-modified TNF receptor superfamily member 2 mRNA, which suppresses the apoptotic pathway and increases leukemia stem cell (LSC) function (57).

#### 3.1.2 CML and m6A

Chronic myeloid leukemia (CML) is typically characterized by the malfunction of monoclonal granulocyte populations caused by the dysregulation of tyrosine kinases on chromosome 22. Although these patients are primarily treated with tyrosine kinase inhibitors (TKIs), TKI resistance remains an urgent challenge. However, little research has been conducted on the association between m6A and CML. PTEN plays a key role in tumor suppression, and the downregulation of PTEN expression can promote leukemia formation in CML leukemia stem cells, which delays disease progression in CML (58). The control of PTEN via METTL3 has also been studied. METTL3 was significantly enriched in the long intergenic noncoding protein (LINC00470). Binding of METTL3 to PTEN mRNA was significantly inhibited in LINC00470-deficient cells, whereas it was markedly enhanced in LINC00470-highly expressed cells (59). Hence, it can be inferred that METTL3 exerts a detrimental influence on the effectiveness of chemotherapy in CML. Furthermore, the METTL3/METTL14 complex is important for CML cell proliferation. METTL3/METTL14 complex inhibitors may be viable therapies for inhibiting tyrosine kinase-resistant CML cells. Ianniello et al. also suggested that the downregulation of METTL3 and METTL14 overcame CML cell resistance (60). Mechanistically, METTL3 maintains ribosome levels and translation by binding to METTL14 in the nucleus to modify nascent transcripts, whose translation is enhanced by cytoplasmic METTL3 localization, suggesting that METTL3/METTL14 complex inhibitors may play a role in CML treatment.

In addition to METTL3/METTL14, RNA m6A readers, IGF2BPs, are implicated in the CML process. Expression of the RNA-binding protein YBX1 was considerably enhanced in CML cells, confirming that YBX1 is necessary for LSC survival. The transcript of the apoptosis-associated gene YWHAZ is stabilized by YBX1 and IGF2BPs in an m6A-dependent manner, and subsequent YBX1 deletion lowers YWHAZ expression by accelerating mRNA decay (61). In conclusion, the aforementioned studies demonstrate the potential mechanisms of m6A modification in CML and provide promising research directions for clinical practitioners.

#### 3.1.3 MM and m6A

Multiple myeloma (MM) is a type of blood cancer caused by the clonal growth of plasma cells in the bone marrow, resulting in anemia, bone pain, and immunosuppression (62). The reader HNRNPA2B1 serves as the m6A’s “on/off” switch. Jiang et al. employed an MTT assay to confirm that overexpression of HNRNPA2B1 induced cell proliferation, whereas its knockdown enhanced cell death. It also enhanced MM development *in vitro* by increasing ILF3-mediated AKT3 expression. Further *in vivo* investigations demonstrated that HNRNPA2B1 increased MM cell proliferation. HNRNPA2B1 identified the m6A site in ILF3 RNA and bind directly to ILF3 to stabilize ILF3 RNA and boost ILF3 production. Specifically, ILF3 binds to and stabilizes AKT3 RNA, causing AKT3 levels to increase and activate the PI3K/AKT pathway (63). To further investigate m6A targets, Zhang et al. performed the m6A-sequence analysis (64), which indicated that knocking out HNRNPA2B1 had an anticancer effect by reducing TLR4 mRNA and protein expression levels.

HNRNPA2B1 is a potential therapeutic target in MM. In addition to the reader HNRNPA2B1, other m6A factors, such as the reader YTHDF2, have been studied. As an independent prognostic factor, YTHDF2 is extensively expressed in MM, and its silencing reduces cell proliferation and the CIP1/WAF1/CDK2-cell cycle protein E1 axis via the EGR1/P21 pathway. Therefore, YTHDF2 has the potential to be a predictive biomarker and prospective therapeutic target in MM (65, 66). Recently, it has been demonstrated that upregulation of METTL3 promotes the proliferation of MM cells. Furthermore, xenograft tumor in nude mice confirmed that METTL3 enhances MM tumor growth through the miR-182/CAMK2N1 signaling axis, suggesting a potential new target for MM therapy (67). Overall, the current researches on the impact of m6A modifications in MM predominantly focus on the reader protein, with limited exploration into the involvement of other factors in MM.

#### 3.1.4 Lymphoma and m6A

Few studies have provided new insights into the role and clinical importance of m6A in diffuse large B-cell lymphoma (DLBCL). According to Kuai et al., WTAP is upregulated in DLBCL tissues and HSP90 can stabilize WTAP at the protein level. These results suggest that HSP90 inhibitors can be used in DLBCL patients with elevated WTAP expression (68). After the antagonist silencing of piRNA-30473, Han et al. (69) observed that patients with significantly elevated piRNA-30473 expression had a poor prognosis. The mechanism of piRNA-30473 is dependent on the increased expression of the methylase WTAP and its key target gene HK2, which plays an oncogenic role in the regulation of cell proliferation and cell cycle in DLBCL (70). Besides, WTAP is engaged in DLBCL cell proliferation and cell cycle regulation (52, 70). WTAP functions as an oncogene in the development of DLBCL (71). However, this study implies that WTAP may regulate DLBCL cell proliferation via downstream CTNNB1. However, the exact mechanisms underlying this regulation remain unclear. Nevertheless, these investigations imply that specific WTAP inhibitors can be used in the clinical treatment of DLBCL. Wei et al. discovered that the lncRNA TRERNA1 was expressed at a higher level in DLBCL tissues than in normal lymph node tissues, implying that TRERNA1 can stimulate cell proliferation *in vitro* and *in vivo* to play an oncogenic role in DLBCL progression. ALKBH5 expression was positively associated with TRERNA1 expression, and both regulated cell cycle progression by controlling P21 protein levels. Thus, TRERNA1 may be useful as a novel lncRNA biomarker for DLBCL (72). In contrast, a study conducted in 2023 revealed that the M6A methyltransferase KIAA1429 was upregulated in patients with DLBCL. The elevated expression of this enzyme is associated with poor clinical outcomes. Mechanistically, KIAA1429 promotes the progression of DLBCL by inhibiting CHST11 expression and attenuating the activation of Hippo-YAP signaling induced by the interaction of CHST11 with the oncogene MOB1B (73).

## 4 m6A modifications in the immunological microenvironment of hematological malignancies

### 4.1 m6A modification and macrophages

Macrophages originating from bone marrow HSCs and monocytes are primarily involved in the recognition, phagocytosis, and degradation of pathogens and tumor cells, which play important roles in tumor development (74). Tumor-associated macrophages (TAMs) are highly plastic in the tumor microenvironment and can be divided into two subtypes with distinct functions: type 1 macrophages (M1) and type 2 macrophages (M2), which play either pro- or anti-cancer roles (75). Emerging studies have focused on m6A alterations in tumors that affect macrophage polarity via various molecular mechanisms. In these investigations, the modulation of macrophage polarization via m6A was mostly focused on METTL3, as opposed to other m6A modifiers such as IGFBP2/3, FTO, and YTHDF2. METTL3 directly methylates the mRNA of the transcription factor STAT1, thereby enhancing mRNA stability and protein expression to initiate M1 macrophage polarization (76). Similar to the above findings, Yin et al. found that the absence of METTL3 hinders the translation of SPRED2, which is mediated by YTHDF1. Through the ERK pathway, SPRED2 promotes the activation of the NF-κB pathway and STAT3 signaling, resulting in a significant increase in the expression of M1-associated genes (TNF-α and IL-6) and M2-associated genes (ARG). Consequently, this leads to the polarization of M1 and M2-like macrophages and increased infiltration of M1/M2-like macrophages and regulatory T cells into tumors, ultimately promoting tumor progression and attenuating the efficacy of anti-tumor immune therapy (77). Furthermore, STAT1 expression was downregulated in M1-polarised macrophages after FTO knockdown, but STAT6 and PPAR expression were lowered in M2-polarised (78). RBM4 interacts with YTHDF2 to degrade m6A-modified STAT1 mRNA, thereby controlling glycolysis and M1 macrophage polarization.

### 4.2 m6A modification and natural killer cells

NK cells are specialized immune effector cells that play an important role in the immunological activation of abnormal cells (79) and the immune modulation through the release of chemokines and cytokines (e.g. RANTES and IFN-γ) (80, 81). Ma et al. previously demonstrated that YTHDF2 is required for IL-15-mediated NK cell homeostasis and survival as well as anti-tumor efficacy (82). Song et al. discovered a favorable association between METTL3 expression and NK cell effector activity. Defects in METTL3 specificity decrease the frequency of multiorgan NK cells and hamper NK cell invasion and effector activity in the tumor microenvironment. Further investigation revealed that in a mouse model, knockdown of METTL3 impaired NK cell reactivity to IL-15 via SH2-containing protein tyrosine phosphatase-2 (SHP-2), boosting tumor growth and metastasis (83). Thus, METTL3 can be regarded as a crucial pillar of NK cell homeostasis and anti-tumor activity.

### 4.3 m6A modification and dendritic cells

Dendritic cells (DCs) are thought to be the commanders of human immune cells that control numerous immune system processes. DCs primarily serve as antigen presenters, acting as messengers to transfer antigenic information to T lymphocytes and stimulate their function (84). Wang et al. discovered in 2019 that methyltransferase METTL3 m6A methylation can promote DC maturation by up-regulating the co-stimulatory molecules CD40, CD80, and the TLR signaling adapter Tirap, TLR4/NF-κB signal transduction resulting in the release of pro-inflammatory factors such as IL-6, TNF-α, and IL-12p70 (85). Han et al. investigated a unique immune escape mechanism in which YTHDF1 enhances lysosomal protease translation while inhibiting antigen cross-presentation (86).

### 4.4 m6A modification and T cells

**Figure 2:**
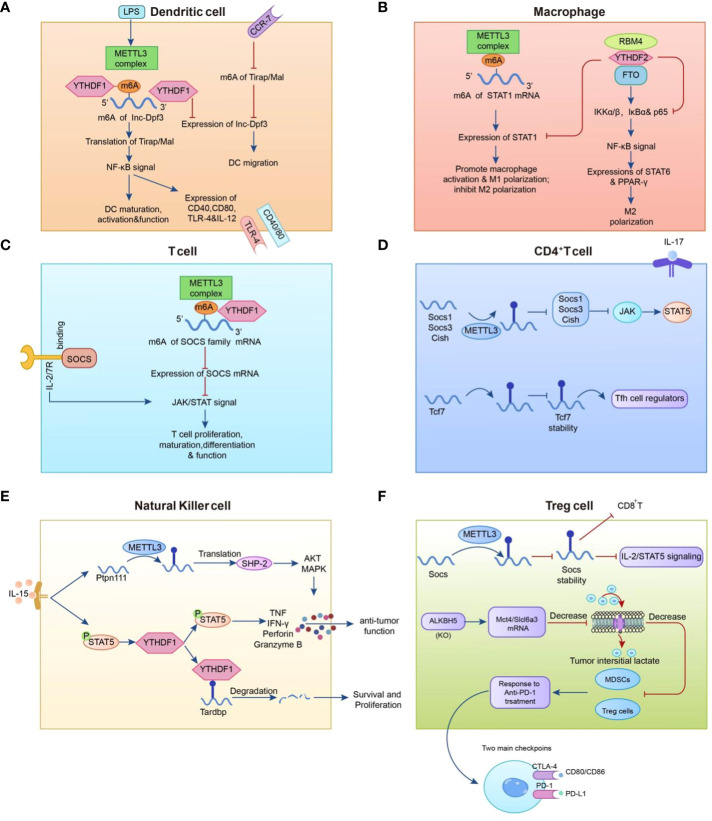
Mechanisms that regulate the m6A modification in immune cells. **(A)** METTL3-mediated m6A methylation enhances dendritic cell (DC) maturation by up-regulating the co-stimulatory molecule TLR signaling adapter Tirap and facilitates pro-inflammatory factor release through TLR4/NF-B signaling in DCs. **(B)** The expression level of METTL3 in macrophages is positively correlated with the methylation modification level of STAT1 mRNA. **(C, D)** METTL3 promotes the degradation of SOCS family mRNAs. **(E)** YTHDF2 augments NK cell secretion of perforin, granzyme B, and IFN- to restrict melanoma metastasis. **(F)** ALKBH5 positively regulates regulatory T cells (Treg), while METTL3 in Treg cells modifies m6A to reduce the stability of Socs mRNAs and participates in anti-PD-1 therapy with myeloid-derived suppressor cells (MDSCs).

T cells, which originate in the thymus and are categorized as CD4 and CD8 T cells based on their cell surface receptors, are a major component of the human immune system (87). METTL3 knockdown in CD4 + T cells caused poor differentiation of naïve CD4 + T cells. Specifically, the IL-7/STAT5 and TCR signaling pathways co-regulate homeostasis and survival of naïve T cells, whereas METTL3 regulates signaling molecules downstream of IL-7. The mRNA levels of the suppressor of cytokine signaling (SOCS) family, an IL-7 signaling inhibitor, were elevated in naïve T cells, and the presence of METTL3 enhanced the degradation of SOCS family mRNAs. Thus, by inhibiting SOCS levels and sustaining IL7 activity, METTL3 can improve naïve T-cell function (88). In the tumor microenvironment, regulatory T cells (Treg) contribute to tumor cell immune evasion by accelerating tumor cell proliferation and infiltration (89). METTL3 depletion was found to inhibit Treg cell function and stability by inhibiting IL-2/STAT5 signaling and promoting cytokine secretion from T effector cells as a means of suppressing Treg cell function and stability for anti-tumor purposes in a study by Tong et al. (90). METTL14 loss decreases naïve T cell development into Treg cells, resulting in elevated levels of Th1 and Th17 and enhanced anti-tumor immunity, as He et al. discovered in a mouse model (91). ALKBH5 also positively regulates Treg cells and is closely related to the ALKBH5 target gene, Mct4/SLC16a3, which is involved in regulating extracellular lactate concentration, Tregs, and the accumulation of polymorphonuclear myeloid-derived suppressor cells (MDSCs) in the TME; increased levels of these substances can lead to the activation of Treg cells, which can diminish the efficacy of anti-PD- 1 therapy (92). This suggests that m6A methylation may be involved in T-cell differentiation and proliferation, potentially influencing tumor growth (**Figure 2**).

### 4.5 m6A modification regulates immune checkpoints

The CD28 family checkpoint protein PD-1 is broadly expressed in a range of cells, including activated T, B, and dendritic cells, and plays several immunomodulatory roles (93, 94). The PD-1 ligands primarily comprise PD-L1 and PD-L2, with PD-L1 expected to be broadly expressed in a range of immune and healthy tissue cells, especially when stimulated by inflammation (95). The binding of PD-1 and PD-L1 causes apoptosis, suppresses T cell proliferation, reduces T cell-mediated immunosurveillance, and assists tumor cells in immunological escape (96). The m6A mutation plays a crucial regulatory role in the PD-1/PD-L1 pathway.

Yin et al. utilized a mouse model to conclude that the deletion of METTL3 reduces the efficacy of YTHDF1-mediated SPRED2 translation, which impedes macrophage reprogramming and reduces the therapeutic effect of PD-1 inhibitors (77). Furthermore, in a study conducted by Han et al., knockdown of YTHDF1, followed by the concomitant use of anti-PD-L1 medicines, improved tumor clearance to 100%. It also provides hope for patients who would otherwise be unable to receive PD-1/PD-L1 antibody therapy (86). YTHDF1 deletion in mice improves the efficacy of PD-1 immune checkpoint inhibitor therapy, implying that YTHDF1 may be a therapeutic target (86).

### 4.6 m6A mutations and tumor treatment resistance

In most patients with hematologic tumors, tumor recurrence often occurs because of resistance to chemotherapeutic drugs. Currently, m6A mutations are linked to medication resistance in a variety of hematological tumors. Despite the availability of numerous chemotherapeutic regimens, extranodal NK/T-cell lymphoma (NKTCL) is a rare and highly aggressive extranodal non-Hodgkin’s lymphoma with an exceptionally poor clinical prognosis (97). Ma et al. discovered that silencing WTAP inhibited dual-specificity phosphatase 6 (DUSP6) at both the mRNA and protein levels, implying that WTAP increased DUSP6 expression in an m6A-dependent manner. Furthermore, as a negative regulator of the ERK signaling pathway, DUSP6 has been linked to treatment resistance in several tumors (98, 99). Similarly, the presence of WTAP increased chemoresistance to DDP in NKTCL cells through a mechanism whereby WTAP lowered the levels of MRP1 and p-glycoprotein (p-gp) in DDP-resistant YTS cells (100). Overexpression of METTL3 in CML lowers the stability of the oncogenic factor PTEN by targeting LINC00470, resulting in the overexpression of p-AKT, suppression of tumor cell autophagy, and chemoresistance. Suppression of LINC00470 expression following METTL3 knockdown restores intracellular PTEN expression (59). Exosomes taken from adipocytes and lncRNAs in them (LOC606724 and SNHG1) reduced chemotherapy-induced apoptosis of MM cells, thereby increasing myeloma cell resistance. Finally, MM cells were shown to improve METTL7A activity via EZH2-mediated methylation of the METTL7A protein, which promotes the packing of lncRNAs into adipocyte exosomes (101). Reportedly, knockdown of circ_0000337 significantly enhanced the susceptibility of drug-resistant MM cells to bortezomib. Moreover, an increase in m6A methylation at circ_0000337-specific sites was observed in bortezomib-resistant cells, leading to enhanced stability of circ_0000337. It was concluded that the m6A level of circ_0000337 and its regulation represent a novel potential therapeutic target for overcoming bortezomib resistance in patients with MM (102). Furthermore, several studies have demonstrated a link between m6A alterations and resistance to AML treatment. According to one of these studies, the overexpression of MEG3 stimulates the production of miR-493-5p, which increases the sensitivity of AML cells to cytarabine (AraC). Furthermore, the deletion of miR-493-5p can boost METTL3 expression, which improves MYC expression and consequently AML cell resistance to AraC (103). Another study suggested that chemoresistance in AML may be linked to AKT. AKT expression was considerably lower in cells overexpressing METTL3, which triggers the PI3K/AKT pathway, promotes MSC adipogenesis, and mediates AML chemoresistance (104). This is supported by the findings in the mouse model of Liao et al. (105). METTL3 also prolongs the half-life of ITGA4 mRNA through m6A methylation, leading to increased ITGA4 protein expression and enhanced homing/implantation of AML cells. Moreover, it contributes to chemoresistance in AML cells (106). Researchers have highlighted the significant role of FTO in the development of drug resistance in AML. FTO regulates AML chemoresistance *in vitro* and *in vivo*. This regulatory effect is mainly based on the FTO-m6A-FOXO3 axis, and FOXO3 is a downstream target of FTO. Hypomethylation of FOXO3 mRNA affects its RNA degradation and further reduces its own expression, which ultimately leads to the weakening of cell differentiation (107). Additionally, FTO overexpression leads to the upregulation of survival and proliferation genes in an m6A-dependent manner, contributing to the development of TKI resistance in leukemia cells (108). Furthermore, a recent study has demonstrated that FTO is significantly upregulated in t(8;21) AML, and there exists a positive regulatory feedback loop between AML1-ETO and FTO. The underlying mechanism involves the upregulation of FTO expression by AML1-ETO through inhibition of PU.1-mediated transcriptional repression of FTO, as well as the promotion of AML1-ETO expression by FTO through inhibition of YTHDF2-mediated decay of AML1-ETO mRNA. Notably, overexpression of IGFBP2 in FTO knockdown t (8; 21) AML cells was found to restore Ara-C tolerance (109). WTAP plays a crucial role in the development of resistance to AML treatment. Knockdown of WTAP in leukemia cell lines enhances the sensitivity of the cells to daunorubicin by preventing the degradation of MYC mRNA in an m6A-dependent manner (110). Notably, another study reached the same conclusion that the upregulation of WTAP leads to increased resistance of AML cells to chemotherapeutic drugs (52). IGF2BP1 upregulation increases leukemia cell resistance to chemotherapeutic drugs by enhancing ALDH1A1, HOXB4, and MYB expression via post-transcriptional regulation (111).

Ubiquitin-specific proteases (USP) have been linked to treatment resistance in T cell acute lymphoblastic leukemia (T-ALL). The authors and their colleagues discovered that ALKBH5 upregulated the expression of USP1 by decreasing m6A levels. Downregulation of ALKBH5 reduces USP1 and threonine kinase B levels, promotes sensitivity to dexamethasone, and reduces CEM-C1 cells (112).

### 4.7 m6A alterations and targeted therapy for hematological malignancies

m6A has been explored as a crucial epigenetic alteration. However, early studies focused on DNA methylation. Azacitidine and decitabine have been approved for clinical use as DNA methyltransferase inhibitors for the treatment of hematologic tumors. Emerging m6A-related drugs for the targeted therapy of hematological tumors have been highlighted in clinical practice in recent years, with an emphasis on FTO inhibitors, METTL3 inhibitors, and IGF2BP2 inhibitors (**Table 1**).

**Table 1:** Specific inhibitors against m6A regulators.

Drug(s)	Targets	Functional mechanism	Phase & Stage	Refs.
R-2HG	FTO	Blocking FTO/MYC/CEBPA pathways and anti leukemia	Preclinical study	[53, 113-115]
FB23	FTO	Inhibiting the m6A demethylase activity of FTO	Preclinical study	[116]
FB23-2 and its analogue 13a	FTO	Up-regulating ASB2 and RARA expression and down-regulates MYC expression	Preclinical study	[117]
CS1/CS2	FTO	Selectively binding FTO and inhibiting its demethylase activity	Preclinical study	[118, 119]
GNPIPP12MA	FTO	Targeting leukaemia primordial cells and inducing ferroptosis	Preclinical study	[120]
SsD	FTO	Direct targeting of FTO increased methylation of the m6A RNA	Preclinical study	[121]
STM2457	METTL3	Direct binding to the METTL3 enzyme and consequent inhibition of its activity	Preclinical study	[49]
STM3006	METTL3	Stimulating cells to induce endogenous interferon responses by forming dsRNA	Preclinical study	[122, 123]
CWI-2	IGF2BP2	Direct binding to the KH4 structural domain of IGF2BP2 competitively inhibits the binding of IGF2BP2 to m6A-modified target RNAs	Preclinical study	[124]
JX5	IGF2BP2	Inhibiting the binding of IGF2BP2 with NOTCH1 deactivates NOTCH1 signaling.	Preclinical study	[125]

Targeted FTO inhibitors are gaining traction in hematological oncology, particularly in the treatment of AML. Currently, researchers have identified the following targeted FTO inhibitors: R-2HG, FB23, FB23-2, and their analogs 13a, CS1, and CS2; GSH-bioimprinted nanocomposites loaded with FTO inhibitors (GNPIPP12MA); GSH-loaded bioblotting nanocomposites (GNPIPP12MA); synthetic compound 11b; and a traditional Chinese medicine extract, Chaihu saponin. R-2HG, an oncogene metabolite, has been proposed as a direct target of FTO that can boost the immune response against tumor cells by lowering FTO expression and disrupting the FTO/MYC/CEBPA pathway (53). Enasidenib (AG-221) and ivosidenib (AG-120) are drugs used to treat relapsed/refractory acute myeloid leukemia (R/R AML). They inhibit mutant IDH2/1, respectively. Interestingly, it has been found that R-2HG can increase the sensitivity of these two chemotherapy drugs (113, 114). R-2HG inhibits leukemia progression by inhibiting aerobic glycolysis and mediating post-transcriptional upregulation of phosphofructokinase platelet (PFKP) and lactate dehydrogenase B (LDHB) expression (115). With a better understanding of FTO, Huang et al. created two synthetic FTO-targeting inhibitors, FB23 and FB23-2, whose mechanisms of action were based on the partial inhibition of FTO m6A demethylase activity. These inhibitors have demonstrated remarkable efficacy against AML progenitor cells in xenograft mice and AML cell lines *in vitro* (116). The researchers then expanded on prior research and created 13a, a newer tricyclic benzoic acid analog with a stronger anti-leukemia cell proliferative action than the previous two. Furthermore, 13a increased ASB2 and RARA expression, while decreasing MYC expression, both of which are major FTO targets in AML cells (117). Su et al. used a virtual screening strategy based on FTO crystal structures from the National Cancer Institute’s Developmental Therapeutics Program (DTP) library of 260,000 small-molecule compounds to identify two compounds, CS1 (Bisantrene) and CS2 (Brequinara), as potential FTO inhibitors. They also concluded that these compounds have potent inhibitory effects on AML cells *in vitro*, particularly in AML cells with high FTO expression. They then showed in a relapsed patient-derived PDX mouse model that they could double the median survival time of AML mice at low doses (118). FTO promotes the expression of immune checkpoint genes, leading to immune escape and rendering AML cells insensitive to activated T cell toxicity. Additionally, FTO enhances LSC/LIC self-renewal, ultimately contributing to the progression of leukemia and resistance to demethylation. The primary mechanism of action of CS1 and CS2 is to selectively bind to FTO and inhibit its demethylase activity. This inhibition led to a decrease in the expression of target genes LILRB4, MYC, and CEBPA by inhibiting YTHDF2-mediated mRNA attenuation. Ultimately, this enhances the cytotoxicity of T lymphocytes, attenuates the self-renewal ability of LSCs, and controls their proliferation. Inhibiting YTHDF2-mediated mRNA attenuation resulted in a lower production of the target genes LILRB4, MYC, and CEBPA mRNAs, which eventually increased T lymphocyte cytotoxicity and immune evasion, weakened LSC self-renewal, and regulated LSCs proliferation (119). The components of Chinese herbs, in addition to these targeted medications, are expected to have effects similar to those of the targeted drugs. In one study, Chaihu saponin D (SsD) was reported to reduce AML cell proliferation and increase apoptosis, both *in vivo* and *in vitro*. SsD specifically targets FTO, increases m6A RNA methylation, and lowers the stability of downstream MYC and RARA transcripts, resulting in the suppression of the associated pathways. Furthermore, one study found that SsD could overcome tyrosine kinase inhibitor resistance in FTO/m6A-mediated leukemia, making it extremely promising. Mechanistic investigations have revealed that FTO-dependent m6A demethylation enhances the stability of mRNA transcripts associated with proliferation and survival, which bear m6A, ultimately leading to increased protein synthesis (121). Furthermore, the synthetic drug 11b is a selective FTO inhibitor that works by increasing the amount of the FTO substrate m6A and driving the upregulation of the FTO target gene MYC, while inhibiting RARA (126). Cao et al. created a bioimprinted nanocomposite material (GNPIPP12MA) that inhibits FTO. GNPIPP12M’s anti-tumor mechanism primarily targets primitive leukemia cells, particularly LSC and triggers ferroptosis by altering the intracellular redox state. GNPIPP12MA also enhances cytotoxic T-cells to promote anti-leukemia immunity, thereby increasing the efficacy of PD-L1 (120).

Kouzarides et al. discovered that STM2457, a METTL3-specific inhibitor, could directly bind to the enzyme and reduce its activity without reducing the activity of other methyltransferases. Further *in vivo* investigations revealed that it can limit the proliferation and development of AML cells and greatly extend the lifetime of mice, with no clear harmful side effects or influence on body weight (49). Subsequently, Guirguis et al. developed STM3006, a second-generation METTL3 inhibitor with high potential, selectivity, and ability to penetrate cells (122). The inhibition of METTL3 stimulates cells to produce endogenous interferon responses through dsRNA formation. Additionally, the antigen-presenting molecule MHC-I was upregulated and expressed in the cell membrane in response to STM3006 stimulation, implying a corresponding enhancement of its antigen-presenting function. The efficacy of the combination of STM2457 and the anti-PD1 antibody was evaluated using a mouse CDX model. The combination significantly prolonged the survival of mice compared with the use of either agent alone. This immunomodulatory mechanism provides an early preclinical scientific basis for combining anti-PD1 immune checkpoint blockade to enhance the anti-tumor effects (123).

Inhibitors that target IGF2BP2 are the third group. CWI-2, discovered by Chen et al., has been verified by both *in vivo* and ex vivo tests. Cellular experiments confirmed that CWI1-2 directly binds to the KH4 structural domain of IGF2BP2 and competitively inhibit IGF2BP2 binding to m6A-modified target RNAs. Furthermore, the feasibility of targeting IGF2BP2 with CWI1-2 as a therapeutic strategy for AML was discovered in a mouse model, and based on the experiments, a combination of chemotherapeutic drugs with CWI1-2 was proposed as an AML treatment (124). Ji et al. tested JX5, a small molecule inhibitor that specifically targets IGF2BP2, and found that it inhibits T-ALL cell proliferation *in vitro* by directly binding to the IGF2BP2 protein and downregulating NOTCH1 expression, and that it significantly delays tumor progression in T-ALL mice *in vivo* (125).

Currently, all the three drug classes are undergoing preclinical studies. Furthermore, Gao et al. suggested that the deletion of METTL3 induces harmful innate immune responses through dsRNA formation, ultimately leading to hematopoietic failure (127). This discovery provides new insights into immunotherapy and serves as a guide for future studies in this field.

## 5 Conclusions

Over the last few decades, immunotherapy has achieved significant success, substantial challenges remain. Resistance to immunotherapy is a leading cause of treatment failure in malignant tumors, and a considerable number of patients do not benefit from it in clinical practice. Tumor immune escape (TIE) is a significant contributor to immunotherapy resistance. This study explored the correlation between m6A and various hematological malignancies as well as currently available m6A modulators for therapeutic interventions. Unfortunately, there is a dearth of clinical trials investigating the efficacy of m6A inhibitors in hematological malignancies; hence, further exploration of their molecular mechanisms is imperative to enhance the effectiveness of specific therapeutic interventions. Although most inhibitors are currently in preclinical trials, it is reasonable to anticipate that the combination of m6A modulators with immunotherapy will emerge as a promising strategy in the near future, addressing the limitations of immunotherapy and enhancing the survival prospects of patients with hematological malignancies.

## Author contributions

SY: Writing – original draft. LX: Supervision, Writing – review & editing. HZ: Writing – review & editing. FL: Writing – review & editing, Funding acquisition, Resources. YL: Writing – review & editing, Funding acquisition, Resources.
